# Supplementary material for: The interactome of intact mitochondria by cross-linking mass spectrometry provides evidence for coexisting respiratory supercomplexes
Source: Mol Cell Proteomics. 2017 Dec 8;17(2):216–32. doi: 10.1074/mcp.RA117.000470 (PMC5795388; doi:10.1074/mcp.RA117.000470)
Supplement: Supplemental Data [file supp_RA117.000470_133922_0_supp_23978_fzffwf.zip › spectra_annotation/mito_spectra_annotation/463-1-11-1-4-1.pdf]

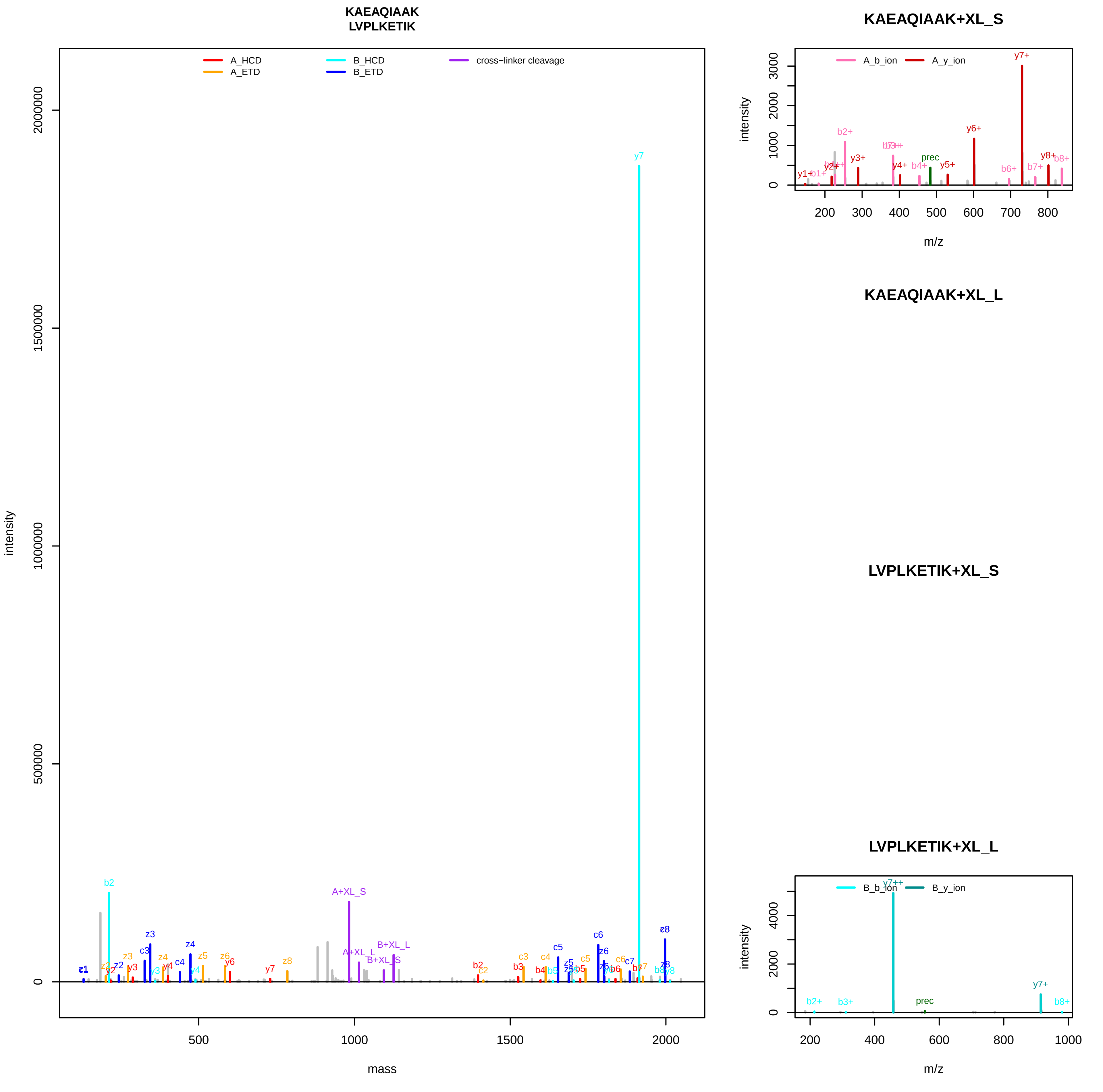
KAEAQIAAK
LVPLKETIK
A_HCD
A_ETD
B_HCD
B_ETD
cross−linker cleavage
intensity
mass
0
500000
1000000
1500000
2000000
500
1000
1500
2000
y7
b2
A+XL_S
A+XL_L
B+XL_L
B+XL_S
KAEAQIAAK+XL_S
A_b_ion
A_y_ion
y7+
y6+
prec
m/z
0
1000
2000
3000
200
300
400
500
600
700
800
KAEAQIAAK+XL_L
LVPLKETIK+XL_S
LVPLKETIK+XL_L
B_b_ion
B_y_ion
y7++
y7+
b2+
b3+
b8+
0
2000
4000
200
400
600
800
1000
